# The long-term clinical outcomes of microvascular decompression for treatment of trigeminal neuralgia compressed by the vertebra-basilar artery: a case series review

**DOI:** 10.1186/s12883-019-1450-z

**Published:** 2019-09-03

**Authors:** Xuhui Wang, Hong Wang, Sha Chen, Hong Liang, Hao Wang, Minhui Xu, Lunshan Xu

**Affiliations:** 1 Department of Neurosurgery, Daping Hospital, Army Medical University, No. 10 Daping Changjiang Street, Yuzhong District, Chongqing, 400042 China; 2 Department of Clinical Biochemistry, Southwestern Hospital, Army Medical University, Chongqing, 400038 China

**Keywords:** Neurosurgery, Microvascular decompression, Trigeminal neuralgia, Vertebra-basilar artery

## Abstract

**Background:**

Microvascular decompression (MVD) is a type of neurosurgery used to treat trigeminal neuralgia (TN) caused by the vertebrobasilar contact/compression. The surgery is not risk-free, however; it may cause recurrent facial pain or other side-effects. The objective of this study was to assess the long-term pain relief and the complications of MVD surgery for the vertebrobasilar compression treatment.

**Methods:**

Twenty-three patients with TN compressed by the vertebra-basilar artery (VBA) were treated with MVD. Teflon felt was placed between the brain stem and the offending artery to mobilize the artery towards the skull base and the clivus. The Barrow Neurological Institute (BNI) Pain Intensity Scale score was used to assess pre- and post-surgical pains.

**Results:**

Of 23 patients with pre-operative BNI IV to V, 19 patients (83%) were pain-free after surgery. Four patients experienced transient partial pain relief with BNI II–III, and 3 of them (13%) were completely pain-free within 3 months. The success rate was 96%. Three patients (13%) had pain recurrences, and one received a second MVD surgery for pain relief during the period of follow-up. Four patients suffered from TN hypesthesia, and only 2 patients (8.6%) had permanent facial hypesthesia, while one patient (4.3%) developed a gradual hearing loss after surgery.

**Conclusions:**

While our success rate of immediate pain relief after surgery was comparable with some reports, the percentage of patients who had pain recurrences was lower, and cases who had permanent facial hypesthesia or developed a gradual hearing loss were fewer after MVD surgery. Our rate of transient complications was higher, and the postoperative pain relief seemed unusually delayed. Our study indicates that MVD is an effective, reliable, and safe neurosurgery for treatment of TN compressed by the VBA albeit our small sample size. Failure of treatment and recurrence of the disease as well as complications could be minimized by preventing displacement of the Teflon implant and extraneous Teflon touching the trigeminal nerves.

## Background

Though most patients with trigeminal neuralgia (TN) do not have vertebrobasilar ectasia (VBE), a small percentage of TN cases may be associated with VBE. Most of these patients with VBE are characterized by significant dilation, elongation, and tortuosity of the vertebra-basilar arteries [1]. Pain results from the compression between the enlarged vessels and the trigeminal nerve at the base of the brain. Although a small dose of antiepileptic/antineuralgic drugs can initially provide excellent pain relief, up to 10–30% of patients do not respond to these drugs and need to undergo surgical treatment [2, 3]. The common surgical treatments for TN include ablative procedures, such as stereotactic radiosurgery and percutaneous rhizotomy, and non-ablative surgical microvascular decompression (MVD). MVD causes little or no facial numbness compared to percutaneous stereotactic rhizotomy and is highly successful as a gold standard first line treatment for TN with a minimal risk of pain recurrence [4, 5].

While MVD neurosurgery has been used to effectively treat TN due to the conflict contact between the trigeminal nerve and the vertebra-basilar artery (VBA), the long-term pain relief and the complications of the surgery need to be determined. The incidence and severity of MVD-associated complications seem largely dependent on the surgeon’s skill and knowledge of local surgical anatomy as well as the size and shape of the implants. In this study, we analyzed the long-term results of MVD treatment in 23 patients whose pre-operative pain scores were IV to V because of VBA compression, and we provided technical tips to minimize pain recurrence and potential complications of the neurosurgery.

## Methods

### Patients and selection criteria

**Fig. 1:**
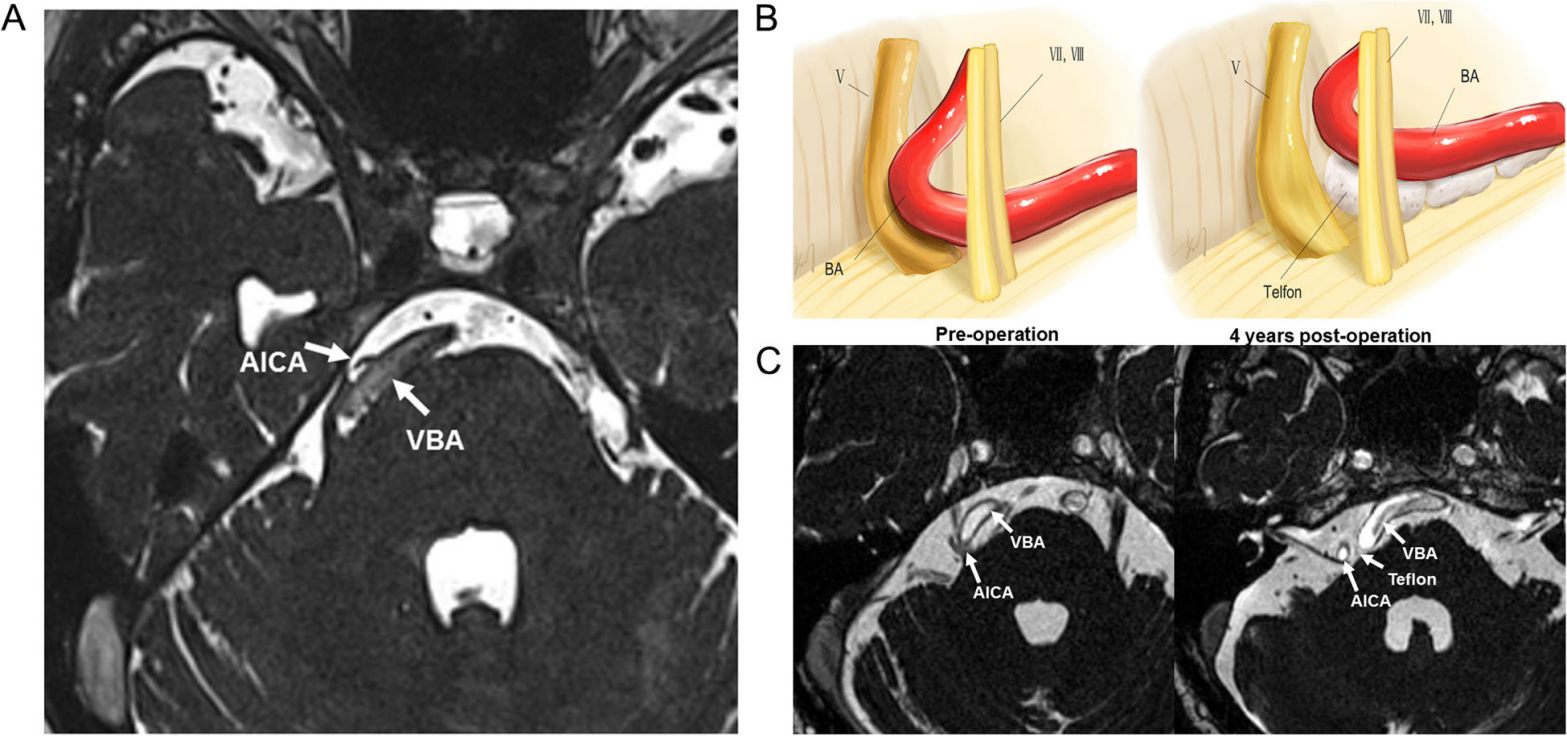
An image of tortuous dolichoectatic vertebrobasilar artery (VBA) and AICA. **a** An image produced by a common 3D-TOF magnetic resonance imaging (MR) shows a tortuous dolichoectatic VBA and the AICA stem were shifted to the right side. The trigeminal nerve is curved and deformed due to compression of the translocated arteries. **b** A schematic diagram of the surgical procedure. Cigarette-like Teflon implants are placed between the VBA and the nerve for decompression. **c** Preoperative (pre-op) and 4 years postoperative (post-op, 4 years) MR images. In pre-op MR, the bilateral vertebrobasilar artery (VBA) is seen to curve towards the right side and compresses the trigeminal nerve as well as the 7th and 8th cranial nerves. The 4 years postoperative image shows that Teflon remains between the VBA and the brain stem without shifting isolated the trigeminal nerve and arteries, but it simultaneously compresses the 5th, 7th, and 8th cranial nerves, which is the cause for recurrence of facial pain

Twenty-three patients with TN associated with the compression of ectatic VBA underwent MVD neurosurgery at the Daping hospital from 2013 to 2016. Before the operation, the patient’s face or skull was examined with 3.0-Tesla magnetic resonance imaging (MRI). A three-dimensional time-of-flight (3D-TOF) sequence was employed with a 2-mm thick slice to elucidate the delicate structures of the trigeminal nerve and blood vessels surrounding the nerve as shown Fig. 1. Patients who met the following diagnosis criteria were selected for surgery and included in the study: 1) severe pain with BNI score IV to V restricted to the trigeminal nerve distribution on one side of the face, 2) TN and VBE diagnosed by MRI, as described previously [1], 3) refractory to pharmacological treatment or development of serious side-effects from the medications, and 4) able to give informed consent and follow-ups. Patients who had known multiple sclerosis, anesthesia dolorosa, or medical contraindications to neurosurgery were excluded. The study was approved by the Ethics Committee of Daping Hospital of Army Medical University of PLA (No. 35(2018)). Formal consents were verbally obtained from all patients, which was also approved by local ethics committee mentioned above.

### Surgical techniques

**Fig. 2:**
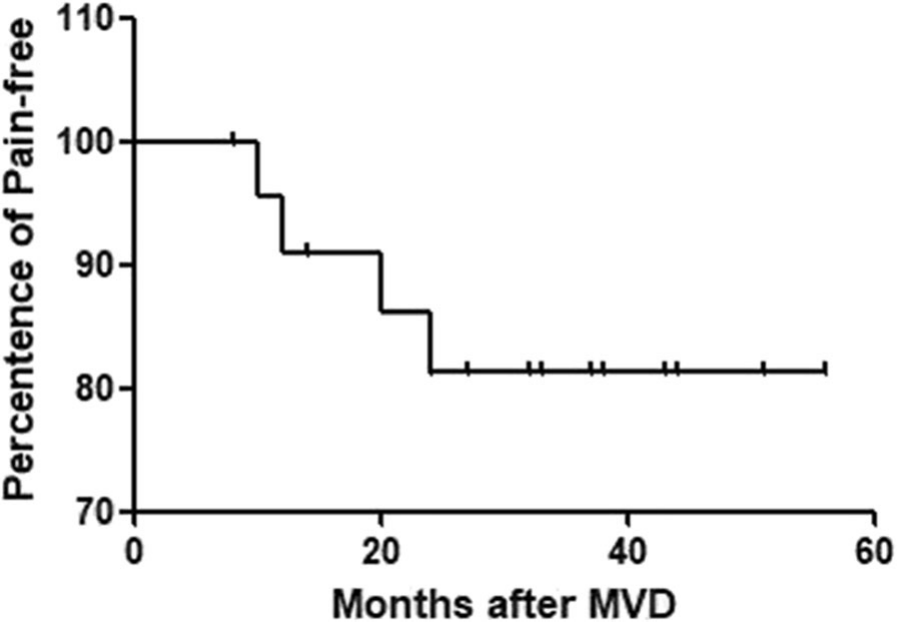
A Kaplan-Meier pain free survival curve. Patients were followed up every 6 months. Follow up time range is 8 to 60 months, with median follow-up time 32 months

A schematic diagram for the procedure is shown in Fig. 2. A mini retrosigmoid craniectomy incision of 3 cm in diameter which is wider than the general MVD keyhole bone window was made to expose the edge of the sigmoidal sinus. Intracranial dissection was conducted from the caudal cranial nerves, as previously described [6]. The arachnoid in cerebellopontine angle (CPA) was opened thoroughly so that the VBA could be raised between the fifth and seventh nerves. Cigarette-like Teflon (polytetrafluoroethene) implants were placed piece by piece between the VBA and the brain stem, gradually pushing the large vessels close to the skull base and away from the root entry/exit zone (REZ). Subsequently, additional Teflon materials were implanted between the nerve and the vessels to prevent the vascular loop from rebounding. Since the nerve was compressed to the tentorium, the SCA and some small veins close or inherent to the nerve were dissected and consolidated to keep the whole nerve free. For the cases whose VBA could not be shifted away from trigeminal nerve completely, Telfon was inserted between the trigeminal nerve and the VBA. No partial or complete nerve section was employed in surgery. Finally, the dura mater was sutured in a watertight pattern with an adaptive dural closure on the outside, followed by closure of the incision.

### Outcome assessment and follow up

The Barrow Neurological Institute (BNI) Pain Intensity Scale score was used to assess preoperative pain and postsurgical outcomes [7]. Postsurgical outcomes were classified as “complete pain relief” (BNI pain score: I), “partial pain relief” (BNI pain score: II–III), or “failure” (BNI pain score: IV–V). The immediate outcomes were assessed as previously reported [8]. Recurrence was defined as a previously relieved pain symptom that recurred at any time during the period of study. Patients were followed up by telephone and periodic clinical checkups for 8 months to 5 years with a median follow-up time of 32 months in this study.

## Results

### Demographic, clinical characteristics and surgical strategy

Out of 411 TN patients who underwent MVD surgery in Daping hospital, 23 were associated with VBA compression, as confirmed by the surgeon during operation, accounting for approximately 6%. The second and/or third division of the trigeminal nerve was commonly compressed while isolated neuralgia resulting from the first division of the trigeminal nerve was not observed in these patients. Detailed demographic and clinical characteristics are shown in Table 1. MR imaging showed a large and tortured VBA in all patients. The VBA generally shifted to the ipsilateral side, and the affected trigeminal nerve was curved and deformed due to compression of the VBA, as shown in a representative case (Fig. 1a). Of 23 cases, 15 patients (65%) were diagnosed with VBE while the SCA and AICA were also found to be compressed near the trigeminal nerve in MR imaging in some cases. In this study, Teflon was used as the only decompressive material without glue or sutures in all 23 patients who underwent MVD surgery (Fig. 1b & c). In two cases (8.7%), the offending VBAs were too large and long. While the arteries could be safely moved toward the petrous bone and clivus and away from the REZ, the distal section of the VBA was pushed to slide along the tentorium. The procedure allowed the offending vessels to remain in contact with the nerve near the access of Meckel’s cave. Teflon felt and gelatin sponges were inserted between the vessels and the nerves with no posterior rhizotomy.

**Table 1:** Summary of clinical characteristics of the patients

Patient characteristics (*n* = 23)	No. of patients (%)
Age (year)	
< 50	1 (4)
50–70	15 (65)
> 70	7 (30)
Sex	
Male	9 (39)
Female	14 (61)
Symptom duration (year) before MVD	
< 2	10 (43)
2–6	7 (31)
> 6	6 (26)
Carbamazepine	
Effective	21 (91)
Ineffective	2 (9)
Previous failed surgery	
No	19 (82)
Thermo- or glycerol-rhizotomy	4 (17)
GSK	2 (8)
Side	
Left	16 (70)
Right	7 (30)
Topography	
V2	7 (30)
V3	2 (9)
V1 + V2	1 (4)
V2 + V3	10 (44)
V1 + V2 + V3	3 (13)
Extent	
1	4 (10)
2	21 (52)
3	15 (38)
Preoperative BNI score	
IV	1 (4)
IV	22 (96)
Symptom characteristics	
Typical	20 (87)
Atypical	3 (13)
Trigger point	
Positive	20 (87)
Negative	3 (13)

### Outcomes

The clinical follow-up periods ranged from 8 months to 5 years with a median follow up time of 32 months. Of the 23 patients, 19 (83%) were completely pain-free immediately after surgery; 4 patients (17%) had partial pain relief, 3 of which (13%) became completely pain-free within 3 months after operation (Fig. 2). The success rate was 96% (22/23). Three patients developed pain recurrence in the second and third years after MVD (Table 2). Of these, one underwent a second MVD neurosurgery in which the position of the offending vessels and Teflon felts were found unchanged, and no new vascular compression was found. Thus, severe adhesion of Teflon implants was determined to be the cause for recurrence. This patient was eventually pain-free but had severe facial hypesthesia/hypalgesia upon removal of the redundant Teflon, and the adhesion was dissected in the second surgery. The third recurrence patient suffered from facial pain and gradual hearing loss within a year after initial surgery. Recent MR imaging revealed that no new vessel compression was seen near the trigeminal nerve, and the Teflon implants remained between the brain stem and the trigeminal nerve, but the material was in contact with REZ, causing a new compression. As shown in Fig. 1c, a large piece of Teflon felt (1.5 cm in diameter) obviously adhered to the 7th and 8th cranial nerve, which might account for the hearing loss in this case. Based on the MRI and his MVD video, we determined that a second decompression surgery would not make enough room to keep the nerve free but would increase the risk of complications. The patient was finally relieved of pain after a retrogasserian glycerol rhizotomy.

**Table 2:** Outcomes of pure microvascular depression for primary trigeminal neuralgia associated with VBA

Patient Assessment	Immediate Postoperative, No. of patients	Follow-up, No. of patients
Postoperative BNI score		
I	19	19
II	3	0
III	1	2
IV	0	1
V	0	1
Neurological deficits		
Hypesthesia	4	2
Facial weakness	2	0
Hearing decrease/deafness	0	1
Cerebellar sign	2	0
Diplopia	1	0

*BNI* Barrow Neurological Institute

Neurological deficits resulting from the surgery were listed in Table 2. Overall, five patients (22%) had neurological deficits. New facial hypesthesia/hypalgesia caused by surgery occurred in 4 patients (17%) and 2 of them developed permanent facial hypesthesia (8.7%). Transient facial weakness, diplopia, and/or cerebellar signs occurred in 4 patients (17%) who recovered from the minor complications within 3 to 10 weeks. Only one patient (4.3%) developed gradual hearing loss.

## Discussion

MVD to separate the offending vessels from the affected trigeminal nerve is the most effective and accepted treatment for TN, and the technique has been reported to have a good success rate. Tatli et al. compared long term effectiveness of all surgical procedures for TN and demonstrated that success rate of immediate pain relief of MVD ranged from 76.4 to 98.2%, with 18.3 to 3% recurrence rate [9, 10]. Despite providing satisfactory results, MVD is not free of complications. Nineteen percent of the patients were reported as having hearing loss, and the mortality rate was 0.37% [10]. Other drawbacks of the MVD includes the recurrence rate, which has been reported to range from 3 to 30%, and implant-induced granuloma, which occurred in 1.3% of patients [10–12]. The great variation of the recurrence and complication rates strongly suggests that the overall outcomes of treatment of TN with MVD surgery might be dependent on the surgeon’s skill and techniques, knowledge of local surgical anatomy, and the types of the wrapping materials in addition to the diagnosis, pre-operation preparation, and disease condition.

In this study, the percentage of the elder patients with TN was greater than in other studies [13]. Consistent with other reports, we also identified that the second and the third divisions of the left trigeminal nerve were more likely involved [14]. While the demographic and clinical characteristics of our patients and our immediate pain-free success rate after surgery were comparable with some reports [4, 14], the percentage of patients who had pain recurrences was lower, and cases that had permanent facial hypesthesia and developed a gradual hearing loss were fewer. We had no patients develop an implant related granuloma. Part of the difference in outcomes found could be explained by the fact that we had more typical patients (87%) as compared with other reports, and patients with a typical pattern of symptoms relapsed significantly less often and were less likely to develop severe complications after surgery [15]. We believe that minimized dissection, macrovascular mobilization and implant insertion between the offending VBA and the nerves in our surgery also contributed to the lower rate of side-effects.

We should point out that our sample size was small, and surgical procedure was in fact at low risk because of the patient selection criteria with relative low threshold. We noticed more complications in our work as the rates of transient complications were a little higher than those for standard MVD. This could be explained by our surgical procedures in which the arachnoid in cerebellopontine angle (CPA) needed to be open thoroughly to expose and mobilize the VBA, including the arachnoid covering cranial nerve VII-XII that should be preserve perfectly to protect these cranial nerves in standard MVD. In addition, MVD operation was done between these nerves, thus retraction was unavoidable. It is possible that these procedures could account for the higher transient postoperative complications. In addition, the postoperative pain relief seemed unusually delayed in this study. However, based on our experience delayed pain relief is commonly seen after MVD. The mechanism is not understood yet. It is generally believed that the treatment course and initial pain types (e.g. typical vs. atypical) may attribute to the delayed pain relief.

In this study, we used the 3D-TOF sequence to visualize the elongated, ectatic VBA and the twisted trigeminal nerve, which helped us determine the location and size of the incision for dissection and reposition of the large vessels in the MVD surgery and observe the position of Teflon implants and the surrounding tissue We prefer to place Teflon felt piece by piece between the VBA and the brain stem, gradually elevating the artery closer to the petrous bone and away from the REZ to decompress the nerve completely, and this approach achieved excellent results. However, we noted that a large volume of Teflon might be inserted into the posterior fossa to push the VBA far away from the trigeminal nerve, which increased the possibility of inflammation, adhesion, and TN recurrence after MVD. As evidenced in this study, we removed the extra Teflon and adhesion in one case and found that the patient’s facial pain immediately disappeared after the second surgery. For two cases whose VBAs were too long and hard, we developed a new strategy. We inserted Teflon between the vessel and the nerve so that the nerve was not completely decompressed as opposed to elevating the vessel away from the brain stem and decompressing the REZ for fear of touching the trigeminal nerve. We found that the insertion procedure was effective for those cases. We believe that distortion of the nerve would not cause TN because the nerve was still distorted after MVD but became relaxed. However, future studies are needed to assess the position and volume of Teflon implants as well as other methods that occupy less space to keep the nerve free and avoid recurrences. In addition, a study with a larger population and a longer follow up time is needed to confirm the findings.

## Conclusions

While our success rate of immediate pain relief after surgery was comparable with some reports, the percentage of patients who had pain recurrences was lower, and cases that had permanent facial hypesthesia or developed a gradual hearing loss or other complications were fewer after MVD surgery. Our study indicates that MVD is an effective, reliable, and safe neurosurgery for treatment of TN compressed by the VBA. Failure of treatment and recurrence of the disease as well as complications could be minimized by preventing both the displacement of the Teflon implant and extraneous Teflon from touching the trigeminal nerves.

## Data Availability

All data generated or analysed during this study are included in this published article.

## Abbreviations

3D-TOF: Three-dimensional time-of-flight
AICA: Anterior inferior cerebellar artery
BA: Basilar artery
BNI: Barrow Neurological Institute
CPA: Cerebellopontine angle
MRI: Magnetic resonance imaging
MVD: Microvascular decompression
REZ: Root entry/exit zone
SCA: Superior cerebellar artery
TN: Trigeminal neuralgia
VA: Vertebral artery
VBA: Vertebra-basilar artery
VBE: Vertebrobasilar ectasia
